# Age-related decrease in collagen proton fraction in tibial tendons estimated by magnetization transfer modeling of ultrashort echo time magnetic resonance imaging (UTE-MRI)

**DOI:** 10.1038/s41598-019-54559-3

**Published:** 2019-11-29

**Authors:** Saeed Jerban, Yajun Ma, Behnam Namiranian, Aria Ashir, Hoda Shirazian, Zhao Wei, Nicole Le, Mei Wu, Zhenyu Cai, Jiang Du, Eric Y. Chang

**Affiliations:** 1 Department of Radiology, University of California, San Diego, CA USA; 2 Radiology Service, VA San Diego Healthcare System, San Diego, CA USA

**Keywords:** Tendons, Diagnostic markers

## Abstract

Clinical magnetic resonance imaging (MRI) sequences are not often capable of directly visualizing tendons. Ultrashort echo time (UTE) MRI can acquire high signal from tendons thus enabling quantitative assessments. Magnetization transfer (MT) modeling combined with UTE-MRI—UTE-MT-modeling—can indirectly assess macromolecular protons in the tendon. This study aimed to determine if UTE-MT-modeling is a quantitative technique sensitive to the age-related changes of tendons. The legs of 26 young healthy (29 ± 6 years old) and 22 elderly (75 ± 8 years old) female subjects were imaged using UTE sequences on a 3T MRI scanner. Institutional review board approval was obtained, and all recruited subjects provided written informed consent. T1 and UTE-MT-modeling were performed on anterior tibialis tendons (ATT) and posterior tibialis tendons (PTT) as two representative human leg tendons. A series of MT pulse saturation powers (500–1500°) and frequency offsets (2–50 kHz) were used to measure the macromolecular fraction (MMF) and macromolecular T2 (T2_MM_). All measurements were repeated by three independent readers for a reproducibility study. MMF demonstrated significantly lower values on average in the elderly cohort compared with the younger cohort for both ATT (decreased by 16.8%, p = 0.03) and PTT (decreased by 23.0%, p < 0.01). T2_MM_ and T1 did not show a significant nor a consistent difference between the young and elderly cohorts. For all MRI parameters, intraclass correlation coefficient (ICC) was higher than 0.98, indicating excellent consistency between measurements performed by independent readers. MMF serving as a surrogate measure for collagen content, showed a significant decrease in elderly leg tendons. This study highlighted UTE-MRI-MT techniques as a useful quantitative method to assess the impact of aging on human tendons.

## Introduction

Tendons are responsible for transferring the mechanical loads generated by muscles to bones. Water may comprise over 60% of the total tendon weight^1^. Of the dry weight of the tendon, 60–85% is composed of collagen, particularly collagen type I, which is highly organized in the form of collagen fibrils, fibers, and bundles of fibers and fascicles^1,2^.

Magnetic resonance imaging (MRI), a non-invasive imaging modality, is routinely used for morphological evaluation of tendons^3–5^. Unfortunately, conventional clinical MRI sequences detect very low signal in tendons, presenting a significant challenge in the accurate assessment of tendons with clinical MRI^6–8^. Specifically, the detected signal intensity of a tissue in MRI depends on various factors, including transverse magnetization relaxation time (T2)^8,9^. Tendons possess very short T2 relaxation times due to a high percentage of an organized collagenous matrix^8^. The low signal-to-noise ratio of tendons might limit differentiation of healthy tendon versus abnormal tissue.

Ultrashort echo time (UTE) MRI sequences are capable of detecting considerable signal of tendons before decay to background levels occurs^8^. Consequently, UTE-MRI has provided quantitative MRI-based assessments of tendons, such as apparent relaxation time of transverse magnetization (T2*), relaxation time of longitudinal magnetization (T1), and magnetization transfer (MT) measurements such as MT ratio (MTR)^6–8,10–16^.

The other critical barrier to the use of clinical MRI and UTE-MRI for quantitative assessment of tendons is the confounding factor of the “magic angle effect.” The magic angle effect is seen in collagen-rich tissues and is due to orientation sensitivity of dipolar interactions of proton nuclear spins^17^. As the tissue fiber orientation approaches 55 degrees relative to the main magnetic field axis, B_0_, frequency changes from dipolar interactions are minimized, resulting in maximized signal intensity^18^. Earlier studies have reported that over 200% of change observed in MR properties of normal tendons and cartilage measured at 3T only resulted from differing orientations of the tissue relative to B_0_^19–22^.

Magnetization transfer (MT) modeling combined with UTE-MRI has recently been used to indirectly measure the macromolecular protons fraction (MMF) in different tissues^20,21,23–28^. Interestingly, two-pool UTE-MT modeling has demonstrated promise as a clinically compatible quantitative technique that is resistant to the magic angle effect^20,21^. Specifically, UTE-MT modeling has been recently implemented on cadaveric Achilles and rotator cuff tendons in order to assess MMF, and showed roughly no orientation angle sensitivity^20,21^. UTE-MT modeling relies on the phenomenon of magnetization transfer to quantify protons, including directly detected water protons (both free and restricted) and indirectly detected macromolecular protons, which possess T2* values too short for direct imaging, even with UTE sequences. This technique provides multiple parameters, including MMF, macromolecular relaxation time (T2_MM_), and exchange rates^20,21^.

Despite the remarkably stable UTE-MT modeling results at different orientation angles, it is currently unclear how sensitive MT parameters are to tendons’ compositional and structural changes through the aging process. If UTE-MT modeling demonstrates significant sensitivity to the aging process in tendon, it may be helpful in the detection of weakened tendon regions and in the prediction of potential tendon injuries.

The main objective of this study was to determine if UTE-MT modeling is a quantitative MRI technique sensitive to age-related changes in human leg tendons.

## Material and Methods

### Subject inclusion

A total of 26 healthy young female subjects (29 ± 6 years old) and 22 elderly female subjects (75 ± 8 years old) were recruited through posted flyers for leg MRI scans. The young healthy subjects were under 40 years old. Elderly female subjects were over 50 years old and in postmenopausal condition. Pregnant women and unhealthy volunteers were excluded after an initial screening questionnaire. No known tendon diseases were present in either cohort.

The institutional review board (IRB) of University of California, San Diego has approved this study. This study was conducted in accordance with applicable good clinical practice requirements and the relevant guidelines and regulations. Written informed consent was obtained from all patients.

### UTE-MRI scanning protocol

The legs in all subjects were imaged using UTE-MRI sequences on a 3T MRI (MR750, GE Healthcare Technologies, WI, USA) using an eight-channel knee coil for both RF transmission and signal reception. Participants were asked to select their desirable leg to be scanned. The imaging slab was centered at the middle of the tibia and localized based on operator experience. To measure T_1_ as a prerequisite for the two-pool MT modeling, an actual flip angle-variable TR (AFI-VTR)-based 3D-UTE-Cones sequence (AFI: TE = 0.032 ms; TRs = 20 ms and 100 ms; FA = 45°; VTR: TE = 0.032 ms; TRs = 20, 80, and 150 ms; FA = 45°; rectangular RF pulse with a duration of 150 µs) was performed with a total scan time of 20 minutes^29^. Additionally, a 3D-UTE-Cones-MT sequence (Fermi saturation pulse power = 500°, 1000°, and 1500°; frequency offset = 2, 5, 10, 20, and 50 kHz; FA = 7°; 9 spokes per MT preparation; rectangular RF excitation pulse of 100µs) was performed for two-pool MT modeling with a total scan time of 14 minutes^30–32^. Field of view (FOV), matrix dimension, nominal in-plane pixel size, and slice thickness were 14 cm, 160 × 160, 0.87 mm, and 5 mm, respectively.

### MRI data analysis

Elastix software (open source software: http://elastix.isi.uu.nl/) was used to register all images to the first T1 image (TR = 20 ms) to compensate for potential subject motion. All scans were smoothed using a Gaussian filter with a 3 × 3 sub-window before T1 and MT measurements.

**Figure 1:**
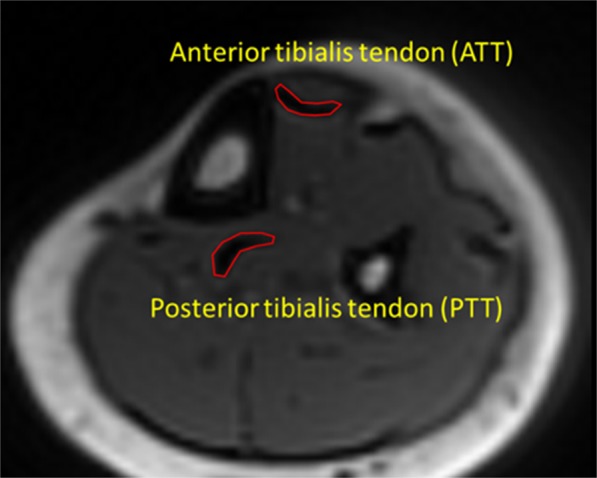
A representative Cones UTE-MRI image from a 50-year-old female subject (TR = 20 ms and TE = 2 ms). Tibialis anterior and posterior tendons were obvious in the MRI images, and are indicated in red contours.

ROIs were selected by three experienced radiology trainees at the anterior tibialis tendon (ATT) and posterior tibias tendon (PTT). Figure 1 shows a schematic of the selected ROIs in a representative Cones UTE-MRI image from a 50-year-old female subject (TR = 20 ms and TE = 2 ms). For quality control purposes, the selected ATT and PTT ROIs by the trainees were overseen and validated by a board certified MSK radiologist for the first three datasets.

T_1_ was measured based on single-component exponential fittings ($S(TR)\propto 1-\exp \,(-TR/T1)+constant$) on the acquired Cones-AFI-VTR data^29^. The acquired data with the set of MT saturation pulse powers (500°, 1000°, and 1500°) and frequency offsets (2, 5, 10, 20, and 50 kHz) were fitted by a modified rectangular pulse approximation (mRP) approach which was previously described^20,23,33^. Super-Lorentzian line shape was used to model the macromolecular proton spectrum. The loss of the longitudinal magnetization of the macromolecular pool was also fitted by a Super-Lorentzian line shape function^23^.

The UTE-MT analysis was performed offline on the acquired DICOM images using an in-house code written in MATLAB (version 2016, Mathworks, Natick, MA, USA). A Levenberg-Marquardt algorithm was employed for the non-linear least-squares fitting in both UTE-MT modeling and T1 fitting within the earlier selected ROIs (Fig. 1).

### Statistical analysis

All statistical analyses were performed using MATLAB software. The differences in T1 and MT modeling results (MMF and T2_MM_) were compared between young and elderly cohorts using a two-sided Wilcoxon rank sum test, which is appropriate for unequal sample size (n = 26 for young cohort and n = 22 for elderly cohort). P-values below 0.05 were considered significant. To investigate the reproducibility of the measurement process, intraclass correlation coefficient (ICC) was measured comparing the T1, MMF, and T2MM in all datasets as estimated by the three independent radiology trainees.

## Results

**Figure 2:**
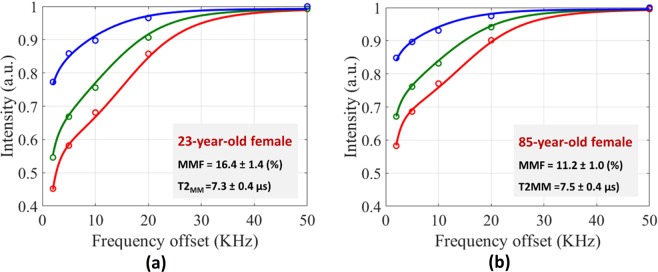
The two-pool MT modeling analyses in the anterior tibialis tendon (ATT) of (**a**) a young female subject and (**b**) an old female subject. Average saturated water signal in selected ROIs are depicted with circles for the three pulse saturation powers (500° in blue, 1000° in green, and 1500° in red) and five frequency offsets (5, 10, 20, 50 kHz). Solid lines are fitting lines based on Super-Lorentzian shape functions. MMF and T2_MM_ refer to macromolecular fraction and macromolecular T2, respectively.

Figure 2a,b show the two-pool MT modeling analyses within the defined ROIs of ATT tendon for a young female subject (23-year-old) and an old female subject (85-year-old), respectively. MT modeling was performed for five off-resonance frequencies and three MT saturation pulse power levels, including 500°, 1000°, and 1500°, which are indicated with blue, green, and red lines. MMF was lower for older subjects in the presented examples.

**Figure 3:**
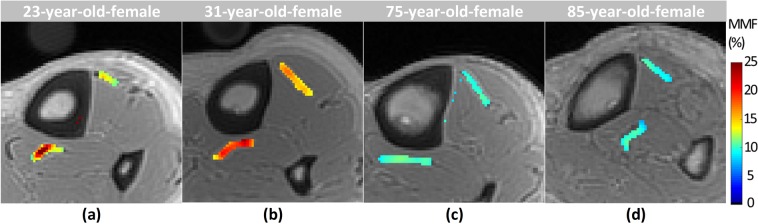
Generated macromolecular proton fraction (MMF) maps for two young healthy subjects, (**a**) 23-year-old female and (**b**) 31-year-old female, and two old subjects, (**c**) 75-year-old female and (**d**) 85-year-old female.

Figure 3 shows the generated MMF maps for two young healthy (23 and 31-years-old) and two old (75 and 85 years old) female subjects. MMF appeared lower in older individuals compared with younger ones for the presented representative results.

Table 1 presents the estimated average and standard deviation values of T1, MMF, and T2_MM_ values in the ATT and PTT among young and old cohorts. Independent measurements by the three radiologists were averaged. ICC between the three independent measurements are also presented in Table 1 for T1, MMF, and T2_MM_ values. For all MRI parameters, ICC was higher than 0.98, which indicates excellent consistency between measurements performed by independent readers. On average, MMF showed lower values for both ATT and PTT in the elderly cohort compared with young cohort.

**Table 1:** Mean and standard deviation of T1 and UTE-MT measurements in anterior and posterior tibialis tendons among young and old cohorts.

		T1 (ms)	MMF (%)	T2_MM_ (µs)
young	ATT	823 ± 156	17.0 ± 5.0	7.3 ± 0.2
	PTT	735 ± 86	21.3 ± 4.8	7.3 ± 0.2
elderly	ATT	789 ± 154	14.1 ± 3.9	7.3 ± 0.3
	PTT	762 ± 123	16.4 ± 3.3	7.1 ± 0.3
ICC		0.984 ± 0.014	0.989 ± 0.009	0.989 ± 0.010

The intraclass correlation coefficients (ICC) were also measured between the three independent readers.

Average percentage differences of UTE-MRI measures in ATT and PTT tendons between elderly and young cohorts, as well as Wilcoxon rank sum test results, are presented in Table 2. MMF demonstrated significantly lower values on average in the elderly cohort compared with the younger cohort at both ATT (−16.8%, p = 0.03) and PTT (−23.0%, p < 0.01) tendons.

**Table 2:** Average percentage differences as well as Wilcoxon rank sum test results in T1, MMF, and T2_MM_ of ATT and PTT tendons between elderly and young cohorts.

	T1	MMF	T2_MM_
ATT	−4.2% (p = 0.51)	−16.8% (p = 0.03)	−0.5% (p = 0.56)
PTT	3.6% (p = 0.67)	−23.0% (p < 0.01)	−2.0% (p = 0.02)

**Figure 4:**
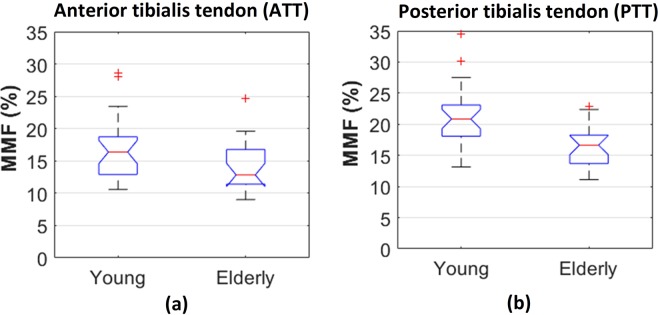
Average MMF in anterior and posterior tibialis tendons for young and elderly cohorts. Average MMF values were significantly lower in tendons of elderly cohort (p < 0.01). The central mark in boxplots indicates the median, while the bottom and top edges of the boxes indicate the 25th and 75th percentiles, respectively. ‘+’ symbol refers to outliers. The elderly cohort showed significantly lower average MMF. The MMF difference between young and elderly cohorts was greater in PTT tendon.

Figures 4a,b show barplots of average MMF values in the ATT and PTT of young and elderly cohorts. As seen in Tables 1 and 2 and in Figure 4, the MMF difference between young and elderly cohorts was greater in the PTT compared with ATT.

## Discussion

UTE-MT modeling acquired with the 3D-UTE-Cones sequence was employed to estimate the age-related differences of collagen proton fraction in human tibial tendons. Aging in general affects the quality, composition, and performance of the tendons^34–37^. UTE-MT modeling as an orientation-insensitive MRI technique and a noninvasive tool to detect collagen fraction changes throughout aging may potentially help in the detection of weakened tendons and in prediction of potential tears or injuries. Timely detection of collagen fraction variation in tendons becomes more crucial due to the age-dependent reduction of tendon’s cellular activities that may impair the tissue repair process.

Aging can affect tendons’ biomechanics directly and lead to weaker tendons as a result of biological changes^34,35,37^. Tendons’ stiffness reduction through aging has been reported from 2% up to 55% depending on the studied tendons^35,36^. Tendons may also adapt their microstructure and composition as a result of decreasing mechanical loads due to weakening muscles and lowering levels of physical activity^35,37^. Age-related changes in muscle strength measuring up to 52% have been reported in the literature^35^. Age-related changes in the cross-section area of tendon have been also investigated, though the variation patterns between different studies were controversial^35,36,38^.

The MMF of both tendons studied here (anterior and posterior tibialis tendons) were significantly lower in the elderly cohort compared with the young cohort (Tables 1 and 2, Fig. 4). MMF reduction most likely indicates a reduction in the collagen content in the studied tendons, although increases in water content can also contribute. The MMF difference in the posterior tibialis tendon was greater compared with the anterior tendon. The PTT plays a key role as a dynamic supporter of the longitudinal arch of the foot, and thusly be affected more intensively by lower activity level in older subjects due to this larger role in physical activity. In a random sample of 1,000 women over 40 years of age, 3.3% had undiagnosed PTT dysfunction^39^. The collagen fibers quality in tendons might not be affected significantly by aging, therefore T1 and T2_MM_ did not show consistent and significant differences in both studied tendons between young and elderly cohorts. Decreases in such relaxation times can indicate a less viscoelastic collagen fiber.

Hodgson *et al*.^40^ used a 2D UTE version of MT modeling and found lower MMF values in the Achilles tendon of a patient with psoriatic arthritis (MMF~16%) compared with healthy volunteers (MMF~ 21%). Ma *et al*. previously used the 2D UTE-MT technique on Achilles tendon specimens and demonstrated the orientation-insensitivity of the UTE-MT modeling, with mean MMF values of 20%^20^. Zhu *et al*. later used 3D Cones UTE-MT modeling to assess rotator cuff tendon specimens and reported significantly lower MMF values for specimens with severe tendinopathy compared with specimens with mild tendinopathy (16% vs. 12%, respectively)^21^. The values of ATT and PTT MMF in our study are in range with these previously reported values^20,21^. However, it is important to note that tendon composition, collagen morphology, and biomechanical properties vary not only between tendons, but in a region-dependent manner within the same tendon^7,13,41^. In young and elderly mice, Wood *et al*. found a substantially increased tangent modulus in the proximal region of the ATT (near the muscle) compared with the rest of the tendon more distally, although all regions of tendons were stiffer with advancing age^41^.

Recently, the 3D Cones UTE-MT modeling has been employed to evaluate MMF in cortical bone organic matrix and to investigate MMF correlations with bone’s microstructural and mechanical properties^24–28^. MMF values reported in cortical bone (~40–70%) have been much higher than tendon due to the much smaller water fraction (~20% bone vs ~60–70% tendon) and corresponding higher fraction of macromolecules.

The first limitation of this study was the relatively long scan time (approx. 34 mins), which may have made it difficult for subjects to remain motionless during the scan. Although, motion was not substantial on the images obtained from most of our subjects, image registration could help the cases where motion occurred between series. Employing different accelerating techniques, such as stretching the readout trajectory, could be used to accelerate the technique and to limit the scans to 20 minutes with negligible resultant errors^42^. Secondly, the fat present in tendon was not taken into account for this study. Intratendinous fat would not be expected to be substantial in the location we imaged, although it may be greater near the enthesis^43^. The potential impacts of fat chemical shift on the estimated MMF values in tendons need to be studied in a future investigation. Reducing the fat signal contamination can be achieved using different fat suppression techniques^44,45^. Thirdly, the presented technique was translated to the leg tendons in healthy young and elderly subjects with no known clinical tendon diseases. However, we cannot exclude subclinical tendinopathy in some cases. Furthermore, our screening questionnaire did not specifically inquire about recent drug therapies. Reversible changes in tendons after ciprofloxacin administration were shown by Juras *et al*.^46^ using both sodium MRI and glycosaminoglycan chemical exchange saturation transfer at 7T. Although special hardware is required for sodium and 7T MRI, future investigations assessing the sensitivity between all of these MRI techniques in different disease conditions should be performed. Fourthly, this study only included female participants that was meant to avoid misinterpretation caused by gender-related differences. According to the literature, female’s tendon structure is different from male’s tendon structure^47^. Moreover, female age-related tendon variation can be different from variations in male cohorts^48^. Nevertheless, performing a similar study on male cohorts and comparing the results with the presented findings on female cohorts will be a future step. This future study will also examine the robustness of the presented sequences and MMF measures on the male cohorts. Fifthly, this investigation only included tibial tendons which their pathology is quite common, in particular involving the PTT^39,49,50^. However other tendons such as patellar and Achilles tendons are also of clinical significance that can be evaluated using the presented MRI techniques in a future well-designed study.

## Conclusion

Two-pool UTE-MT modeling was investigated for its capability to assess age-related collagen fraction differences in human leg tendons. MMF obtained from MT modeling, as an index for collagen fraction in tendons, showed significantly lower (~20%) values in anterior and posterior tibialis tendons of elderly subjects compared with young subjects. This study highlighted UTE-MT MRI techniques as useful methods to assess tendons in the aging population, which may be used in the future for detection of weakened tendons and prediction of potential injuries.
